# Prediction of Liquid Magnetization Series Data in Agriculture Based on Enhanced CGAN

**DOI:** 10.3389/fpls.2022.929140

**Published:** 2022-06-17

**Authors:** Jing Nie, Nianyi Wang, Jingbin Li, Yi Wang, Kang Wang

**Affiliations:** ^1^College of Mechanical and Electrical Engineering, Shihezi University, Shihezi, China; ^2^Key Laboratory of Modern Agricultural Machinery of Xinjiang Production and Construction Corps, Shihezi, China

**Keywords:** series data, liquid magnetization, data enhancement, prediction, biological effect of magnetic field

## Abstract

The magnetized water and fertilizer liquid can produce biological effect of magnetic field on crops, but its residual magnetic field strength is difficult to be expressed quantitatively in real time, and accurate prediction of it is helpful to define the scope of action of liquid magnetization. In this paper, a prediction model for liquid magnetization series data is presented. It consists of conditional generative adversarial network (CGAN) and projected gradient descent (PGD) algorithm. First, the real training dataset is used as the input of PGD attack algorithm to generate antagonistic samples. These samples are added to the training of CGAN as true samples for data enhancement. Second, the training dataset is used as both the generator and discriminator input of CGAN to constrain the model, capture distribution of the real data. Third, a network model with three layers of CNN is built and trained inside CGAN. The input model is constructed by using the structure of two-dimensional convolution model to predict data. Lastly, the performance of the model is evaluated by the error between the final generated predicted value and the real value, and the model is compared with other prediction models. The experimental results show that, with limited data samples, by combining PGD attack with CGAN, the distribution of the real data can be more accurately captured and the data can be generated to meet the actual needs.

## Introduction

In recent years, with the emergence of large-scale time-series data and the improvement of computing power, time-series data prediction has become increasingly important in many fields, such as weather (Hewage et al., 2020; Karevan and Suykens, 2020), energy consumption (Divina et al., 2019), financial indicators (Zhang et al., 2019), retail (Beheshti-Kashi et al., 2015), medical monitoring (Xia et al., 2022), anomaly detection (Munir et al., 2018), and traffic prediction (Li et al., 2015). The deep neural network shows great potential in mapping complex non-linear feature interaction. When processing time-series data, it can automatically learn the time correlation of time series and directly adapt to the data without any prior assumptions. It has been proved that it can successfully solve the problem of time-series data prediction (Sen et al., 2019; Wan et al., 2019).

Time-series data are a series of data indexed by time dimension. This kind of data describes the measured value of a measured subject at each time point in a time range, that is, a one-dimensional corresponding relationship is constructed between the data and time nodes. If the range of time-series data is extended, the data collected on different time nodes will be extended to the data collected on different detection nodes. This detection node is a one-dimensional physical variable that plays a core role in the detection data, that is, a series of physical quantities with the same rhythm as the time node, such as magnetic field strength, voltage, and current. These physical quantities can reflect the change trend of detection data in dimension. In this way, many machine learning models applied to time-series data prediction will be able to predict a wider range of series data, such as prediction and deployment of physical and chemical parameters (Nie et al., 2021a; Wang et al., 2022). This will help to improve the ability of data mining based on the series data.

The biological effect of magnetic field refers to the dominant or recessive effect on the growth and metabolism of organisms under the action of magnetic field environment (Nyakane et al., 2019; Radhakrishnan, 2019). In the process of agricultural production, the integrated management and distribution of crop water and fertilizer supply can be realized by using the integrated irrigation equipment of water and fertilizer. The steady-state controllable magnetic field can be generated by energizing, and the mixed liquid of water and fertilizer with different residual magnetic field strength can be induced by changing the magnetic field parameters. The influence of magnetic field is applied to crops through drip irrigation equipment, and then, a series of magnetic field biological effects are produced to improve the yield and quality of crops (Nie et al., 2021b). The residual magnetic field strength of water fertilizer mixed liquid magnetized by magnetic field is the fundamental factor affecting the biological effect of crop magnetic field. Therefore, this paper hopes to predict the magnetization series data of water and fertilizer mixed liquid, so as to lay a foundation for further clarifying the degree of biological effects of magnetic field on crops.

The residual magnetic field strength of liquid magnetization is affected by the magnetic field strength of magnetization space, magnetization time, the change of permeability caused by different water, fertilizer ratio, and other factors. The ratio of water and fertilizer required by crops in different growth periods is determined. When the water and fertilizer liquid with a certain flow rate passes through the magnetized space, the magnetization time is the same. Therefore, the influence of the magnetic field strength of magnetization space on the residual magnetic field strength of the liquid magnetization is the core factor. The liquid magnetization series data have the same characteristics as other time-series data. The mapping relationship is as follows: The residual magnetic field strength data map the change data we are concerned about, such as weather temperature, retail sales, and energy consumption, and the magnetization spatial magnetic field strength maps the time series. All series data are faced with such a problem, that is, limited by the equipment and conditions, the data samples obtained are limited. How to carry out in-depth data mining on this basis is a problem worthy of exploration.

The application of machine learning in data mining of series data needs to solve the problem of few data samples, while the few-shot learning method does not rely on large-scale samples training data and can be quickly generalized to the new task containing only a small number of supervised information samples, avoiding the high cost of collecting data (Li and Chao, 2020; Wang et al., 2020; Li and Yang, 2021; Yang et al., 2022). Few-shot learning has many excellent algorithm models in image classification (Liu et al., 2018; Li et al., 2020) and text classification tasks (Yan et al., 2018). In addition, using few-shot learning for tasks such as oral comprehension (Kumar and Baghel, 2021), image extraction (Li et al., 2021), and disease diagnosis (Zhong et al., 2020; Li and Chao, 2021a) also has good generalization ability. The idea of learning with a small amount of data to get a good effect model can greatly improve the ability of in-depth learning in the field of agricultural production and has achieved good results in some applications where data samples are not easy to obtain or sample size is small, such as pest control (Li and Yang, 2020), crop counts, and positioning (Karami et al., 2020). At the same time, how to balance between sample quantity and sample quality has also attracted the attention of researchers. Li et al. proposed an effective indicator of distance entropy to distinguish the quality of sample data from the perspective of information and through an embedding range judgment (ERJ) method in the feature space (Li and Chao, 2021b,c; Li et al., 2022). It is confirmed that good data with a small number of choices can achieve the same performance as all training data.

How to optimize the prediction model with reasonable data expansion methods when the sample data are limited is a problem that needs to be solved. Few-shot learning based on data enhancement improves the diversity of samples. In the case of less series data, generating effective samples according to the existing few-shot data and data enhancement of target domain samples has become a new solution direction. Generative adversarial network (GAN) is one of the most promising data enhancement driven algorithms for unsupervised learning in complex distribution in recent years. Through the mutual game learning of generative model and discriminant model, it can produce quite good output, which shows the great potential of inferring physical phenomena. Although GAN has achieved some success in generating data, it still has defects such as mode collapse and unstable training. In order to solve these problems, scholars put forward an improved method of adding antagonistic attack to GAN. Liu and Hsieh proved that the integration of GAN and antagonistic attack can enhance each other (Liu and Hsieh, 2019). By adding antagonistic attack to GAN, such as projection gradient descent (PGD) attack, it can not only improve the defense success rate of discriminator against samples, but also accelerate the network convergence speed and guide the training of better generators. Because the data generation method of the original GAN is too free, it cannot fully capture the distribution of real data and cannot produce data that fully meets the needs. Therefore, conditional generative adversarial network (CGAN) is formed after adding conditional constraints on the basis of the original GAN. CGAN can be seen as an improvement of turning unsupervised GAN into a supervised model. Under the guidance of conditional constraints, it can improve the quality of generated data, so as to better meet the needs of researchers.

In this paper, aiming at the prediction of residual magnetic field strength of water and fertilizer liquid, when the series data samples are limited, PGD antagonistic samples are added to the training of CGAN as true samples for data enhancement, and a prediction modeling method based on CGAN data enhancement is proposed. First, the real training dataset is used as the input of PGD attack algorithm to generate antagonistic samples, and these samples are added to the training of CGAN as true samples for data enhancement. Then, the training dataset is used as the condition input of generator and discriminator of CGAN at the same time to constrain the model, capture the real detection data distribution, and improve the discriminator's ability by antagonistic samples, so as to improve the generator's ability to generate real data. Finally, the network model of three-layer convolutional neural network (CNN) inside CGAN is established and trained. The structure of two-dimensional convolution model is used to construct the input model to predict data. The performance of the model is evaluated by the error between the final generated predicted value and the real value, and the model is compared with other prediction models. This study can provide some implications for prediction of series data in future with limited samples.

## Materials and Methods

### Dataset

**Figure 1:**
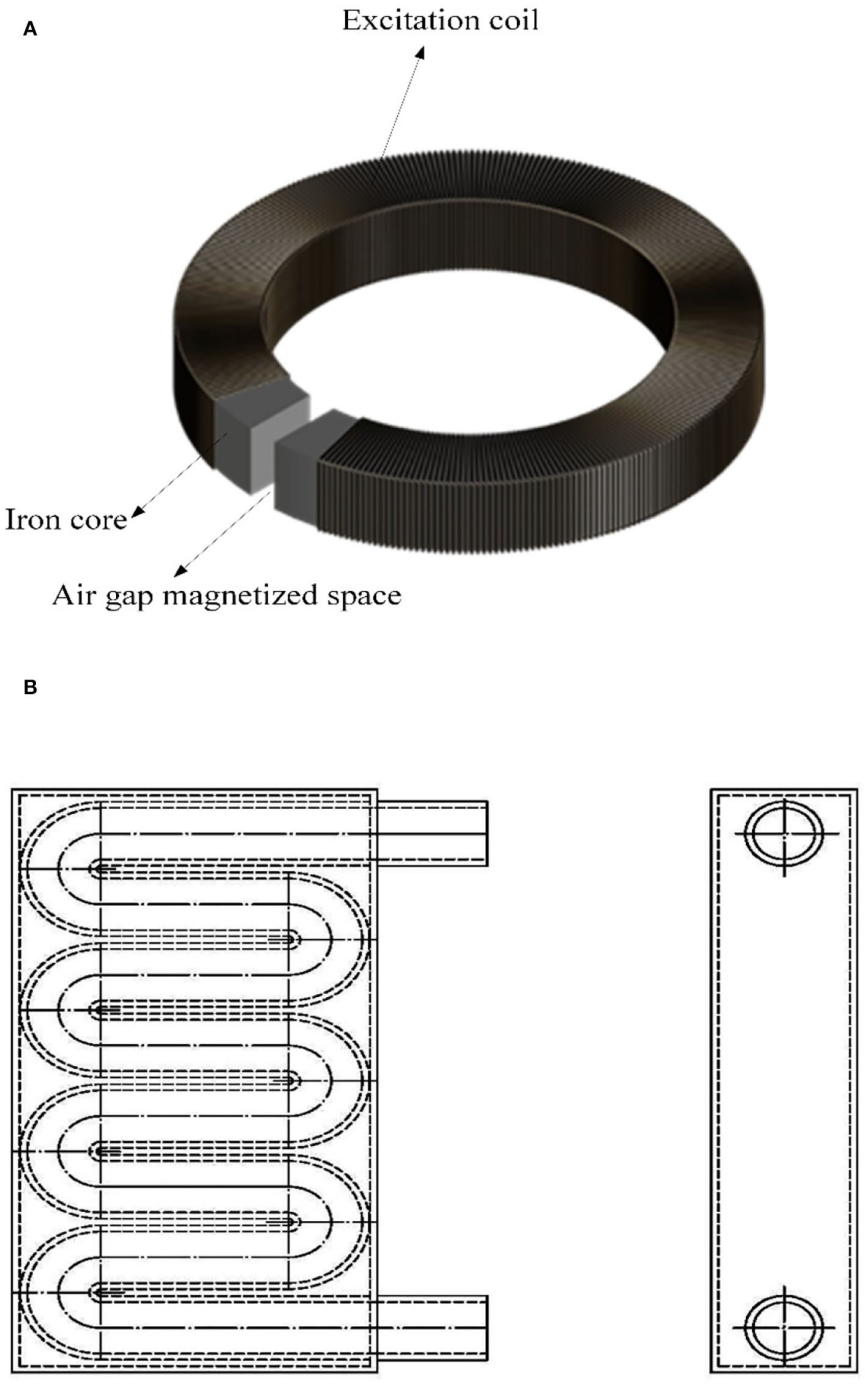
**(A)** Liquid magnetizer structure. **(B)** Liquid magnetized space pipeline.

The training and test datasets used in this study are based on the data of residual magnetic field strength after magnetization of water and fertilizer liquids with different flow rates collected on the liquid magnetization test platform. It consists of a magnetizer and a liquid magnetization space pipeline. The structure of liquid magnetizer consists of iron core, excitation coil, and air gap magnetization space as shown in Figure 1A. The iron core structure is annular, the material is cold-rolled non-oriented silicon steel B50A470, the excitation coil uses circular copper-coated wire, the insulation grade is F, the nominal diameter is 1.50 mm, the reserved air gap magnetization space size is 100^*^40^*^200 mm, and the excitation mode is DC adjustable excitation. To fully magnetize the liquid as it passes through the air gap magnetized space, the liquid flow through the magnetized space pipeline is designed as a closed S-shaped composite pipe structure, which is placed in the air gap magnetized space as shown in Figure 1B. When the liquid is magnetized, it flows in from the upper tube opening and out from the lower tube opening.

There are many input variables that affect the magnetization effect of liquids. Ignoring the influence of secondary variables, the magnetic field strength in the air gap magnetization space and liquid velocity are selected as the main variables that affect the magnetization effect of liquids, and the residual magnetic field strength of the liquid magnetization is selected as the index to measure the magnetization effect. When collecting test data, adjust the current of excitation coil by adjustable DC regulator power supply, change the intensity of magnetic field in the air gap magnetization space, measure the magnetic field strength at the center point of the air gap magnetization space by Tesla meter, measure the flow rate by placing a velocity meter at the water inlet of the liquid magnetization space pipeline, set a sampling hole at the fixed interval of the water outlet, collect the magnetized liquid, and measure the residual magnetic field strength by Tesla meter.

In order to improve the generalization of the model and avoid modeling within local data intervals, data are collected within the magnetization working interval of the liquid magnetizer by uniform sampling. By adjusting the excitation current of the magnetizer, the residual magnetic field strength series data at the same distance can be obtained for liquids with different flow rates, assuming that there are *n* liquids with different flow rates passing through the liquid magnetized space pipeline. For the flow rate of *v*_*i*_ liquid after magnetization by a magnetizer, under the action of *j* different air gap magnetization space magnetic field strength, there is *n* set of detection data, which is recorded as $D_{i}={\left\{x_{i,j}\right\}}_{i=1}^{n}$.

### Methods

#### CGAN

GAN is a data modeling algorithm that generates a set of samples from the data probability distribution *p*_*data*_, providing a way to learn deep representation without a lot of training data. This method, proposed by Goodfellow et al. (2015), has rapidly become a research hotspot in recent years. It is also one of the most interesting ideas in the field of machine learning in recent years. The most successful applications of GAN are image processing and computer vision, such as image super-resolution (Bulat et al., 2018), image synthesis and manipulation (Dong et al., 2018; Wang et al., 2018), and video processing (Liao et al., 2019). GAN processes series data by learning the probability distribution of a given dataset and generating synthetic data that conforms to the same distribution. Therefore, it can synthesize seemingly real artificial data. The application of GAN to series data mainly focuses on discrete issues, such as text generation tasks (Xu et al., 2018), and in a continuous state, GAN is used to generate auditory data (Liu et al., 2021). In addition to these data types, there are also some attempts to apply GAN, such as medical time-series data generation (Kiyasseh et al., 2020), wind speed probability prediction (Cheng et al., 2018), and composite time series in smart grid (Zhang et al., 2018). However, there are few studies on the application of GAN to the probability prediction of sensor detection data. In fact, the detected data have the same mapping relationship as the time-series data. Based on the limited data of residual magnetic field strength of water and fertilizer liquids, this paper uses GAN method to predict its change trend.

GAN is an unsupervised learning method that improves the quality of generated data by learning through a game between generator and discriminator. This competitive approach no longer requires a hypothetical data distribution, but uses a distribution to sample directly, thus truly approaching the real data in theory, which is the greatest advantage of GAN. However, this way of entering a random vector and getting a resulting object is too free for us to control what kind of object is produced. Therefore, CGAN is formed when conditional constraints are added to the original GAN. CGAN is an extension of GAN, which allows us to put models on some extra information *y*. This information can be any type of ancillary information, such as category labels or data from other modes. CGAN achieves conditional constraints on the generated object by conveying additional information *y* to the discriminant model and the generated model as part of the input layer.

CGAN contains two “confrontation” models: Generation Model to capture data distribution and Discrimination Model to estimate the probability that a sample will come from real data instead of synthetic samples. Generator inputs two objects at the same time, a sample *z* sampled from a prior distribution and a conditional *y*, which outputs the resulting data $\tilde{x}=G\left(z|y\right)$. To learn how to generate distribution *x* on real dataset *p*_*data*_(*x*), noise vector *z* is sampled from known prior distribution *p*_*noise*_(*z*) (usually Gaussian or uniform), the generator takes *z* as input, and it also needs to sample from conditional *y* to generate sampled data whose distribution follows *p*_*data*_(*x*). A mapping function *G*(*z*|*y*) is constructed from *p*_*noise*_(*z*) and *y* to data space. In the generation model, a priori input noise *p*_*noise*_(*z*) and conditional information *y* form a joint hidden layer representation. Discriminator also has two inputs, conditional *y* and generator generating data $\tilde{x}$, and outputs a scalar that represents a score $D:D\left(\tilde{x}|y\right)\to \left(0,1\right)$. This score evaluates two things: whether $\tilde{x}$ is real or not, and whether $\tilde{x}$ and *y* match. Models G and D are trained simultaneously: fixed discriminant model D, adjusted G parameters to minimize log(1−*D*(*G*(*z*|*y*))) expectations; fixed the generation model G and adjusted the parameters of D to maximize the expectations of $\log D\left(\tilde{x}|y\right)+\log\left(1-D\left(G\left(z|y\right)\right)\right)$. This optimization process can be boiled down to a “minimax two-player game” problem with the objective function as shown in Equation (1):


(1) $$\begin{array}{l} \underset{G}{\min}\underset{D}{\max}V(D,G)=E_{x\sim p_{data}(x)}[\log D(x|y)]\\ \;+E_{z\sim P_{noise}(z)}[\log(1-D(G(z|y)))] \end{array}$$

where **E** represents mathematical expectation, *x* represents the sample of sampling distribution *p*_*data*_(*x*) in real data, which corresponds to the collected training set data in this study, *z* represents the noise sampled in a priori distribution *p*_*noise*_(*z*), *D*(*x*|*y*) represents the probability that the discriminant model judges that the real data are true data under the constraint of condition *y*, and *D*(*G*(*z*|*y*)) represents the probability that the discriminant model judges that the data generated by random noise *p*_*noise*_(*z*) and condition *y* is true data, and *V*(*D, G*) represents the value function of the discrimination model and the generation model. In an ideal state, it is expected that the value function of the discrimination model will obtain the maximum value and the value function of the generation model will obtain the minimum value.

**Figure 2:**
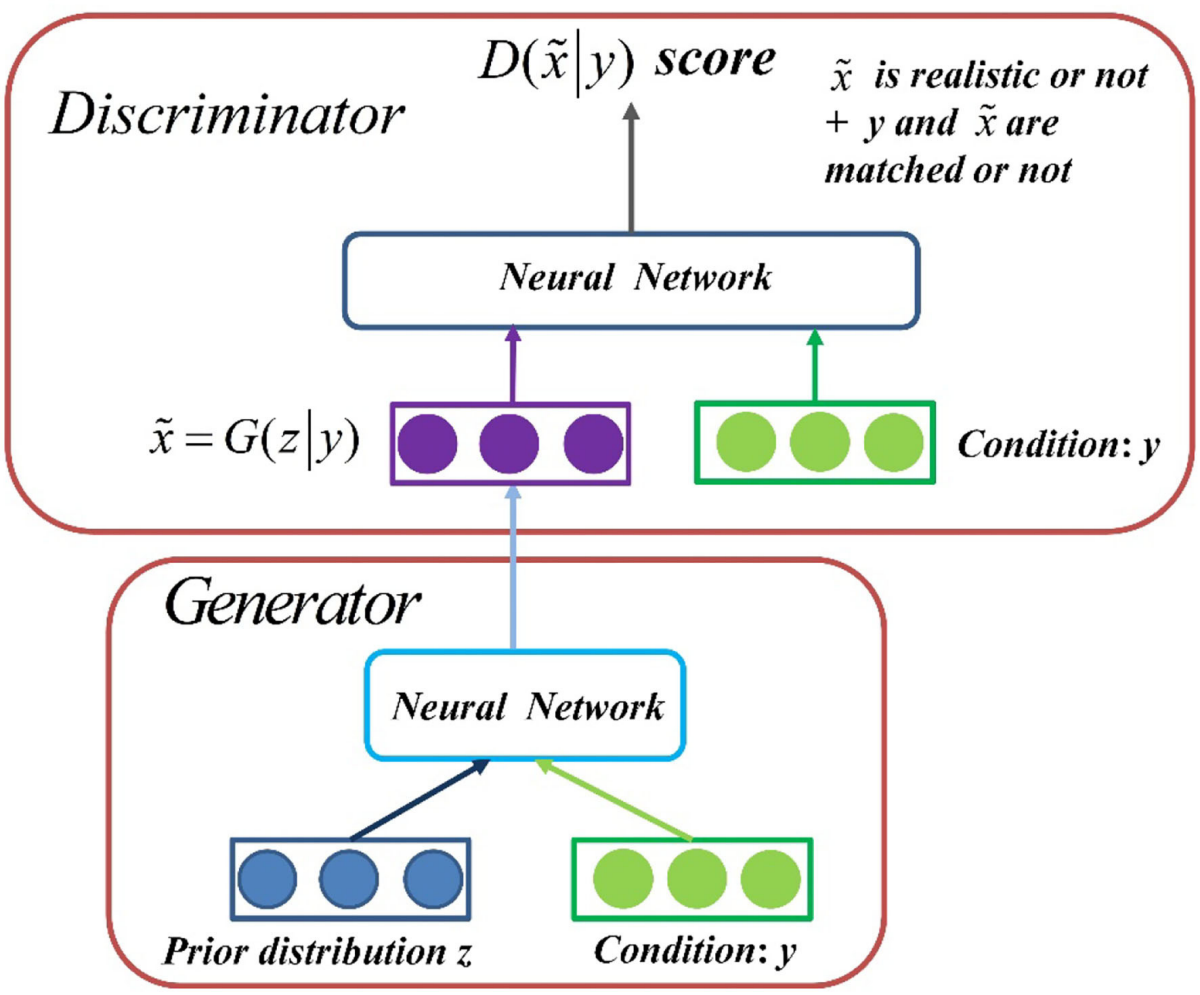
Network architecture of CGAN.

The network architecture of CGAN is shown in Figure 2. The generator takes the sample *z* and condition *y* sampled from the a priori distribution as the input to generate data $\tilde{x}$. The discriminator inputs both condition *y* and generated data $\tilde{x}$ into a network to get a score to measure whether the object $\tilde{x}$ is true and whether it matches the constraint *y*.

#### Project Gradient Descent Attack

The concept of confrontation attack comes from the input samples formed by adding subtle disturbances to the samples, which makes the network output wrong prediction. The basic idea of counter training is as follows:


(2) $$\begin{array}{l} \underset{\theta }{\min}E_{\left(x\right)\sim D}\left[\underset{r_{adv}\in S}{\max L\left(\theta ,x+r_{adv}\right)}\right] \end{array}$$

The inner meaning in Equation (2) to find a group of antagonistic samples in sample space *D* that maximizes the loss function *L* and the countermeasure sample *x* is obtained by the combination of the original sample and the disturbance term *r*_*adv*_ obtained by some means, and the disturbance term *r*_*adv*_ is in disturbance space *S*. Outer meaning: facing the countermeasure sample set composed of such a group of samples, the expected loss of the model on the countermeasure sample set should be minimized by updating the model parameter θ.

Confrontation learning has achieved good results in the image field (Chen et al., 2019), and this way of confrontation attack training can be transferred to series data. How to add disturbance to the confrontation attack is a thorny problem in the prediction of series data. PGD attack algorithm is improved on the basis of fast gradient method (FGM) algorithm. Its essence is to find the optimal disturbance through multiple iterations. Therefore, it is very suitable for discrete non-linear series data model. In this study, the antagonistic samples are generated through the algorithm. On the basis of real data, the disturbance in confrontation *r*_*adv*_ is optimized and calculated through multiple iterations of PGD attack algorithm. The calculation process is shown in Equation (3).


(3) $$r_{adv}:=\underset{r_{adv}\in S}{\arg\max}L(f(\theta ;x+r_{adv})$$

where *f* represents the network parameterized by weight θ, and *L* represents the loss function. The purpose Equation (3) is to find disturbance *r*_*adv*_ to maximize the loss value of a point *x*_*adv*_: = *x*+*r*_*adv*_, so that this point is most likely to become the antagonistic sample. Specifically, it is to carry out forward and backward propagation again and again, calculate loss in forward, calculate parameter grad in back, calculate disturbance *r*_*adv*_ according to gradient again and again, and add new disturbance *r*_*adv*_ to the gradient of embedding layer again and again. If it exceeds a range, it will be mapped back to the given range. Finally, the gradient calculated in the last step is accumulated on the original gradient, that is, the original gradient is updated by accumulating the gradient corresponding to the disturbance in step *t*. The multiple iterative process against disturbance *r*_*adv*_ is shown in Equations (4) and (5).


(4) $$r_{adv/t+1}=\prod {\left\|r_{adv}\right\|}_{2}\leq \varepsilon (r_{adv/t}+\alpha g(r_{adv/t})/{\left\|g(r_{adv/t})\right\|}_{2}$$

(5) $$\;\text{g}({\text{r}}_{\text{adv}/\text{t}})=\nabla_{{\text{r}}_{\text{adv}}}\text{L}(\text{f}(\theta ,\text{x}+{\text{r}}_{\text{adv}/\text{t}}))$$

where ||_*r*_*adv*_||2_ ≤ ε is the constraint space of the disturbance, α is the small step size, and ∏ means to perform projection on ε−*ball*. If the disturbance amplitude is too large, pull the origin part back to the boundary of *ball* to ensure that the disturbance is superimposed in *ball* for many times after multiple operations.

In this study, the antagonistic samples generated by PGD attack algorithm are used as the expansion of the original dataset and added to the model training together, which is equivalent to a way of data expansion. The purpose of confrontation attack training is no longer to defend against gradient based malicious attacks, but more as a kind of regulation to improve the generalization ability of the model.

### CGAN Data Enhancement Model Framework

#### Overall Framework

In this paper, PGD attack algorithm is combined with CGAN. Aiming at the magnetization series data of irrigation water and fertilizer mixture, under the condition of limited data samples, a prediction model using CGAN to enhance the data based on the original data is constructed. The original sample data are attacked by PGD attack algorithm to generate antagonistic sample data. The PGD antagonistic sample is added to the training as a true sample to enhance the data, improve the defense success rate of antagonistic samples, and enable CGAN to generate series data closer to the distribution of real samples.

**Figure 3:**
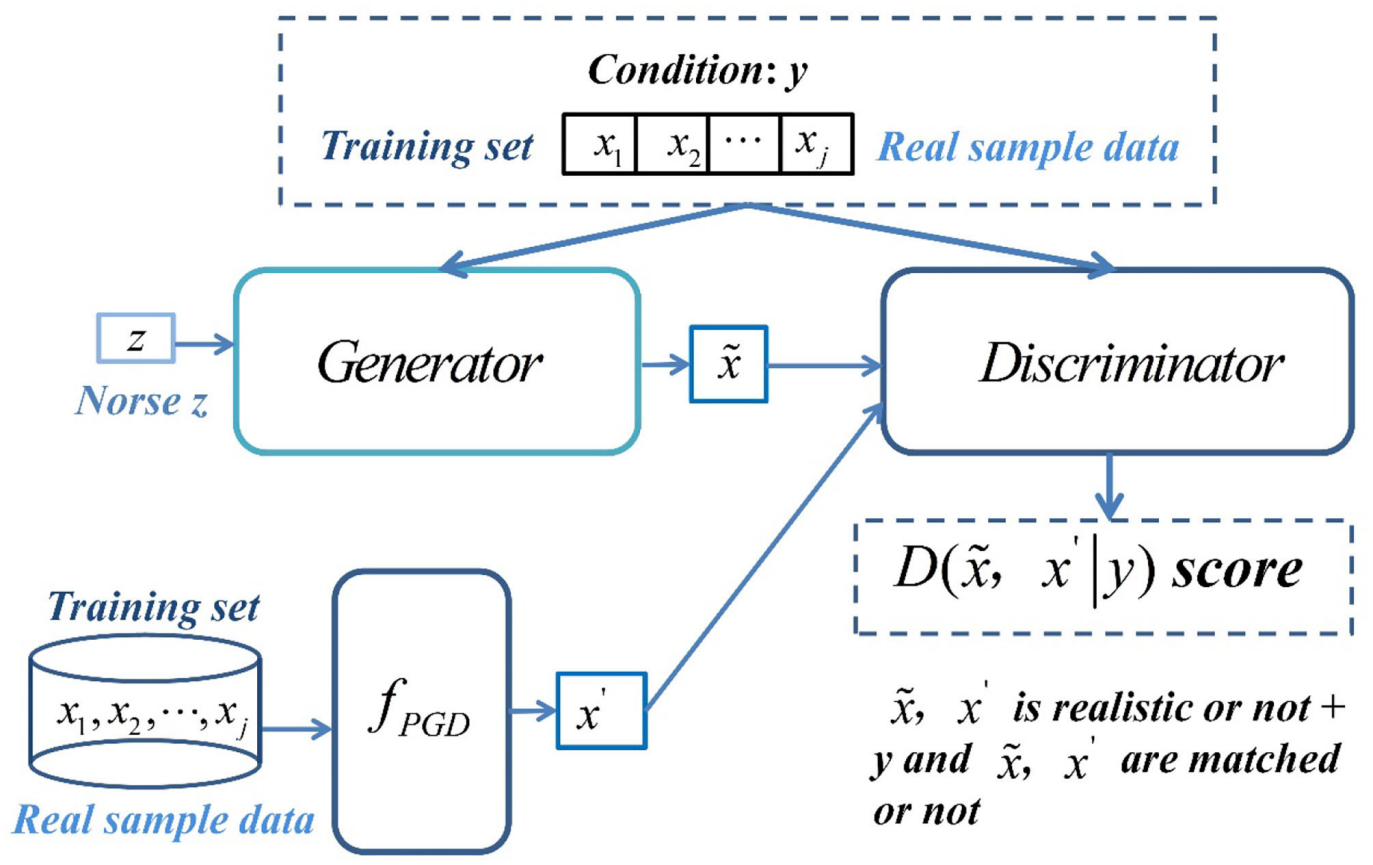
Overall framework of CGAN data enhancement model.

CGAN data enhancement model is mainly composed of generator, discriminator, attack algorithm *f*_*PGD*_, and conditional dataset *y*. Its overall framework is shown in Figure 3. The *f*_*PGD*_ algorithm is responsible for generating antagonistic samples. Its training dataset is completely the sampled real sample data. The generated antagonistic samples are used as the extended training dataset of discriminator. The work of generator is to convert random noise into realistic data with semantic information, and the work of discriminator is to distinguish whether the input data are true as much as possible.

The overall objectives of the model are as follows: The random noise vector *z* is given in the generator, and the training dataset containing real sample data is used as the input of conditional dataset *y* to generate conditional constraints and the generator generates synthetic sample $\tilde{x}$. The training sample, that is, the real sample (*x*_1_, *x*_2_, …, *x*_*j*−1_, *x*_*j*_), is used as the input of the PGD attack algorithm to generate the antagonistic sample *x*′. The discriminator receives the synthetic sample $\tilde{x}$ and the antagonistic sample *x*′ at the same time and takes the condition *y* as the judgment basis to judge whether $\tilde{x}$ and *x*′ are true and match the condition *y*. The generator and discriminator are trained alternately under the guidance of discrimination loss to gradually enhance the ability of discrimination antagonistic samples, so as to make the sample data generated by the generator more real.

#### Generator and Discriminator Network Framework

In this paper, convolutional neural network (CNN) is introduced to construct the internal structure of generator and discriminator. CNN has the powerful ability of multi-hidden layer feature extraction. The introduction of CNN can improve the stability, convergence speed, and data quality of CGAN, because the samples of one-dimensional convolution model are easy to over fit during training, and the anti-noise performance of one-dimensional convolution model is not as good as that of two-dimensional convolution model. In order to enhance the generalization ability and robustness of the whole model, the structure of two-dimensional convolution model is used to construct the input model. Therefore, it is necessary to transform the one-dimensional series data into two-dimensional equivalent information degree and arrange it into two-dimensional form of *n*×*n* for convolution, which is more conducive to model feature extraction. In order to ensure the integrity of sliding sampling of two-dimensional convolution kernel, the input data and output data form an *n*×*n* matrix. The random noise and conditional data are spliced into an *n*×*n* matrix and input into CNN. In order to enable the network to learn more suitable spatial sampling methods independently, the spatial pooling in CNN is not used, and the step convolutions are used to enable the network to sample in the autonomous learning space. Batch normalization is used between levels to accelerate convergence and slow down over fitting, so as to make the gradient propagation deeper. In the output layer, tanh activation function is used, and the other layers are activated by ReLU. Finally, the network generates prediction data.

**Figure 4:**
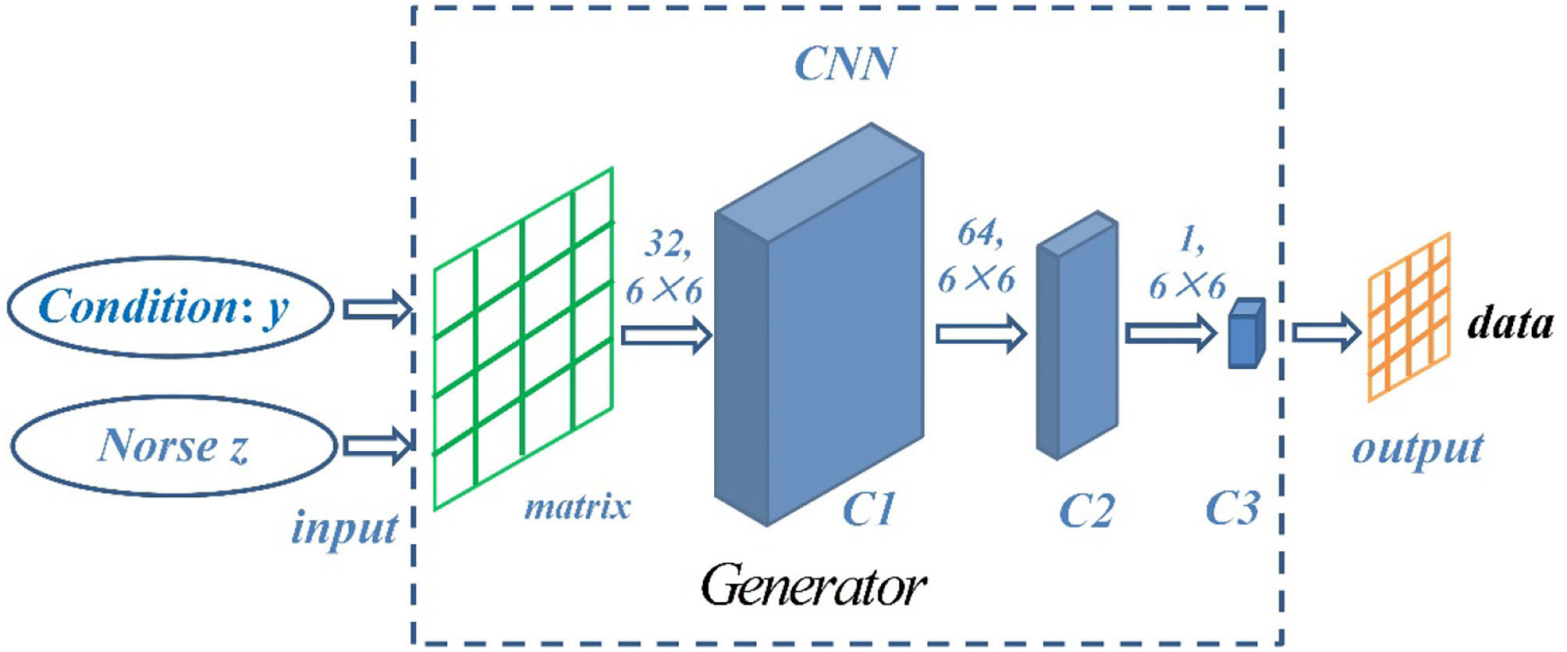
Network structure of generator.

The generator is composed of three layers of CNN, and its structure is shown in Figure 4. The random noise and conditional data are spliced into a matrix and then input. The convolution layer C1 is obtained by convolution of 32 6 ×6 convolution cores, the convolution layer C2 is obtained by convolution of 64 6 ×6 convolution cores, and the convolution layer C3 is obtained by convolution of 1 6 ×6 convolution core to obtain the prediction data and output. The sliding step size is set to 2.

**Figure 5:**
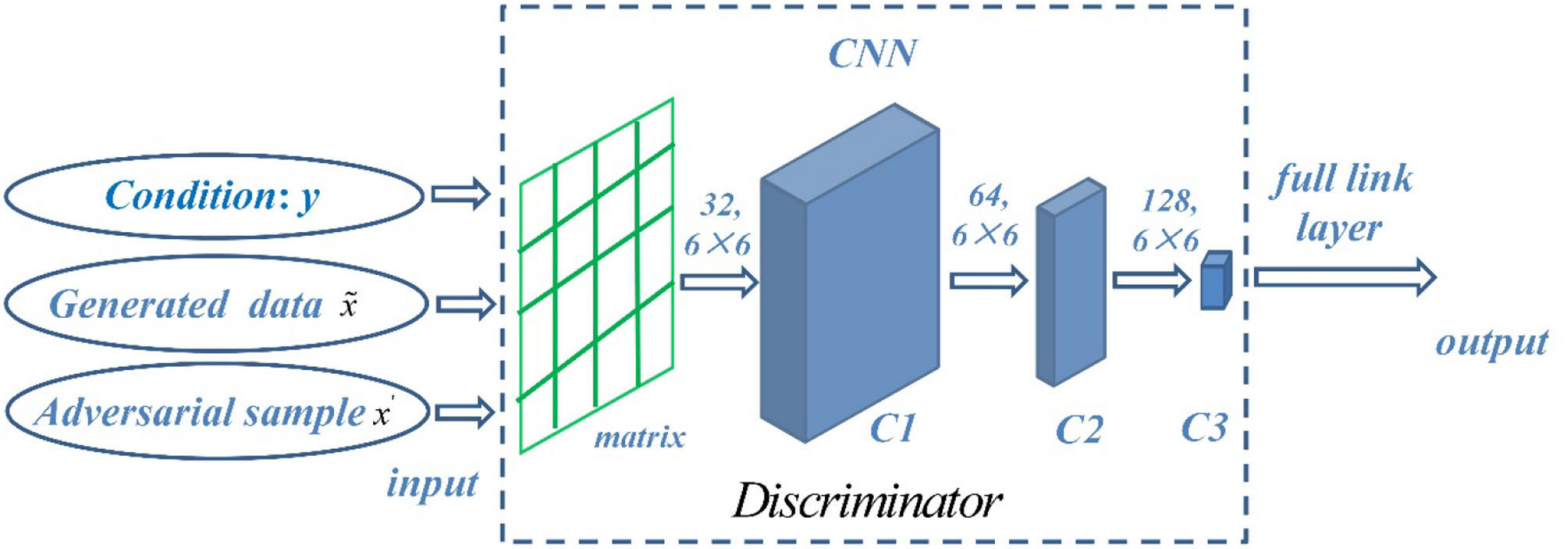
Network structure of discriminator.

Discriminator splices conditional data, generated samples, and antagonistic samples into a matrix as the input of convolution layer. The discrimination model is composed of three layers of CNN, and the hidden layer of the discrimination model uses Leaky ReLU function as the activation function. Finally, the full link and sigmoid activation function are used to judge the true and false, so that the result is mapped between (0, 1). In the discrimination model, convolution layer C1 is obtained by convolution of 32 6 ×6 convolution cores, convolution layer C2 is obtained by convolution of 64 6 ×6 convolution cores, convolution layer C3 is obtained by convolution of 128 6 ×6 convolution cores, and the sliding step size is set to 2. Finally, the full link outputs the discrimination result, and its structure is shown in Figure 5.

When training CGAN, the generation model and discrimination model are trained alternately. In the process of training the generation model, the weight value of the generation model is set according to the deviation between the prediction data generated by the generation model and the real data, the condition deviation, and the discrimination result of the discrimination model. In the process of training the discrimination model, the conditional data, the data generated by the generation model and the antagonistic samples, need to be input into the discrimination model. The discrimination model needs to judge whether the input data are the probability of the real detection data conforming to the conditional distribution and update its own parameters according to the discrimination deviation. In the training process, the random gradient descent algorithm is used to update the discriminant model once and then update the generated model.

### Algorithm

First, initialize parameter θ_*d*_ of discriminator and parameter θ_*g*_ of generator, fix generator, and make discriminator learn. Several vectors are randomly sampled from a Gaussian distribution or uniform distribution. At the same time, *m* samples are randomly selected from the conditional dataset *y* and input into the generator to obtain the corresponding generated data and input into the discriminator. At the same time, the training samples, that is, the real samples, are generated by the *f*_*PGD*_ attack algorithm and sent to the discriminator to improve the generalization ability of the model. Discriminator also randomly selects *m* samples from the conditional dataset *y* to learn how to identify the real data and the generated data, give high scores to the real data and low scores to the generated data as much as possible, and update the parameters of discriminator by regression task in the training process. The algorithm of learning discriminator is shown in Table 1.

**Table 1:** Algorithm of learning discriminator.

**Step 1. Learning discriminator**
1. Input real sample training datasets(*x*_1_, *x*_2_, …, *x*_*j*−1_, *x*_*j*_),$x^{{}^{\prime }}\leftarrow f_{PGD}\left(x\right)$;
2. Sample *m* noise samples from a distribution, such as a Gaussian or uniform distribution,{*z*_1_, *z*_2_, ···, *z*_*m*_};
3. Random sampling of *m* samples from conditional dataset *y*,(*x*_1_, *x*_2_, …, *x*_*m*−1_, *x*_*m*_);
4. Get data $\left\{{\tilde{x}}_{1},{\tilde{x}}_{2},\cdot \cdot \cdot ,{\tilde{x}}_{m}\right\}$ generated by G, where $\tilde{x}=G\left(z_{i}|y\right)$;
5. Randomly select *m* generated data $\left\{{\tilde{x}}_{1},{\tilde{x}}_{2},\cdot \cdot \cdot ,{\tilde{x}}_{m}\right\}$ and *m* antagonistic samples $\left\{{x^{{}^{\prime }}}_{1},{x^{{}^{\prime }}}_{2},\cdot \cdot \cdot ,{x^{{}^{\prime }}}_{m}\right\}$;
6. Randomly select *m* conditional samples (*x*_1_, *x*_2_, …, *x*_*m*−1_, *x*_*m*_);
6. The objective function is recorded as *V*, and θ_*d*_ is updated by maximizing (gradient rise) *V*, that is, θ_*d*_←θ_*d*_+η∇*V*(θ_*d*_). The objective function *V* is as follows: $V=\frac{1}{m}\overset{m}{\underset{i=1}{\sum }}\log D\left(y_{i},x_{i}\right)+\frac{1}{m}\overset{m}{\underset{i=1}{\sum }}\log\left(1-D\left(y_{i},{\tilde{x}}_{i}\right)\right)+\frac{1}{m}\overset{m}{\underset{i=1}{\sum }}\log\left(1-D\left(y_{i},x_{i}^{\prime }\right)\right)$

Then, fix the discriminator and adjust the parameters of the generator. The goal is to enable the data generated by the generator to deceive the discriminator and get high scores as much as possible. The algorithm of learning generator is shown in Table 2.

**Table 2:** Algorithm of learning generator.

**Step 2. Learning generator**
1. Sample *m* noise samples from a distribution (such as Gaussian distribution or uniform distribution),{*z*_1_, *z*_2_, ···, *z*_*m*_};
2. Random sampling of *m* samples from conditional dataset *y*,(*x*_1_, *x*_2_, …, *x*_*m*−1_, *x*_*m*_);
3. The objective function is recorded as *V*, and θ_*g*_ is updated by maximizing (gradient descent) *V*, that is, θ_*g*_←θ_*g*_−η∇*V*(θ_*g*_). The objective function *V* is as follows: $V=\frac{1}{m}\overset{m}{\underset{i=1}{\sum }}\log\left(1-D\left(G\left(y_{i},{\tilde{x}}_{i}\right)\right)\right)$

## Results

### Experimental Data Preparation

In this study, simulating the integrated management process of cotton water and fertilizer, drip irrigation pipeline network is laid out with one film, two tubes and four rows, and main, branch and capillary network structure. The water and fertilizer liquid magnetizer is placed at the head of the capillary network. The capillary outer diameter is ϕ20*mm*, and the capillary flow is $1.4L/h\tilde{\;}2.8L/h$. There will be differences in different growth stages of cotton, and the corresponding liquid flow rate is $4.46m/s\tilde{\;}8.92m/s$. In the process of data collection, the flow rate of liquid was set as $4m/s\tilde{\;}9m/s$, increasing by 1*m*/*s*. The data of residual magnetic field strength after water and fertilizer liquid passing through the magnetized space pipeline at different flow rates were collected in *n* = 6 groups. Each group of data was sampled from $100mT\tilde{\;}880mT$ to 20*mT* with uniform increment of gap magnetized space magnetic field strength. There were *j* = 40 data samples in each flow rate group, and *N* = *n*×*j* = 240 series data were obtained. The water–fertilizer fusion liquid ratio is 1000*kg* water with urea (containing *N*46%)50*kg*, calcium superphosphate (containing *P*_2_*O*_5_64%)25*kg*, potassium sulfate (containing *K*_2_*O*50%) 25*kg* added.

The dataset is divided into training set, test set, and query set, and there is no intersection among these three parts. The training set selects data for measuring residual magnetic field strength of water and fertilizer liquids at a flow rate. The test set selects another test data at a different flow rate. To fully compare the performance of the prediction models and other models proposed in this study, the query set will select the detection data at different flow rates than the training set and the test set. In order to verify the correctness and generalization of the proposed model for predicting the magnetic field strength of water and fertilizer liquids, training set, test set, and query set are divided twice on the dataset, and the details are shown in Table 3.

**Table 3:** Partition of datasets.

**Split mode**	**Training set**	**Test set**	**Query set**
	**Liquid flow rate (40 samples)**	**Liquid flow rate (40samples)**	**Liquid flow rate (40 samples)**
Split-1	4*m*/*s*	5*m*/*s*	7*m*/*s*
Split-2	6*m*/*s*	8*m*/*s*	9*m*/*s*

In two different segmented datasets, we use the training dataset as the conditional dataset for the entire model. It consists of 40 conditional data, all of which are true test series data, which constrain the data generated by generator and serve as the basis for discriminator to determine whether the input data are true and the data distribution is reasonable. The training dataset is also used as input to the *f*_*PGD*_ attack algorithm to generate antagonistic samples.

**Figure 6:**
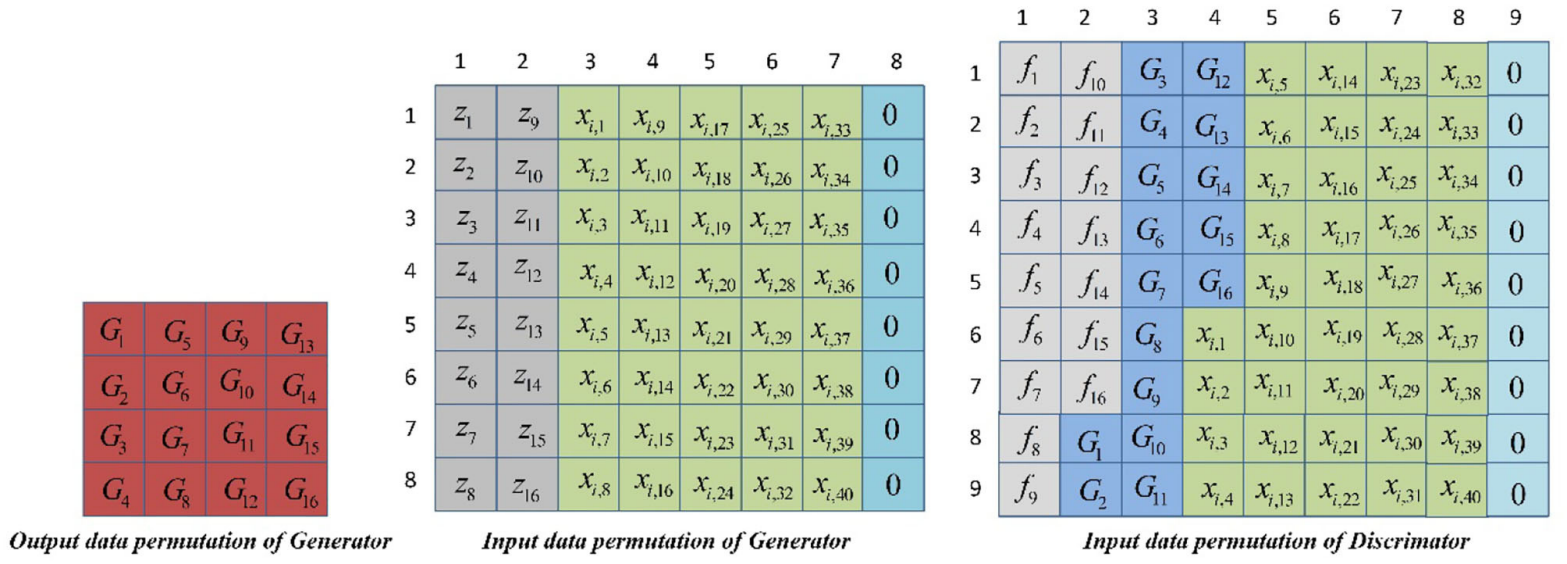
Data arrangement of generator and discriminator.

The input data to generation model are conditional data and noise data, since conditional data are a segmented training dataset, including 40 series data at a set of flow rates. The generated model will generate 16 prediction data based on the input conditional velocity data, which form the matrix of 4 ×4. To maintain the correspondence between the noise data and the output data of the generated model, 16 random noise data are generated by the function as input. The generated model splices 16 input noise data and 40 conditional data, expands the edge data, and fills in eight “0” at the end to form 8 ×8 input matrix. To maintain the correspondence with the data generated by the generation model, the *f*_*PGD*_ attack algorithm also generates 16 antagonistic samples on the basis of each set of training data. The discrimination model splices 16 antagonistic samples generated by the *f*_*PGD*_ attack algorithm, 16 predictive data generated by Generator, and 40 conditional data and fills in nine “0” at the end to form the 9 ×9 input matrix. The purpose of generator and discriminator input data expansion is to fill in the required input matrix. In this study, the input matrix is expanded with “0,” or with the last data value or mean. Expansion of edge data has no effect on the extraction of sample features. The prediction errors and accuracy of the models with different filling modes are compared in Table 4. Generator's generated data, input data arrangement, and discriminator's input data arrangement are shown in Figure 6.

**Table 4:** Comparison of prediction error and precision of models under different filling methods.

**Filling method**	**ȳ_*MAE*_/*mT***	**ȳ_*RMSE*_/*mT***	**ȳ_*FA*_/*%***
Filling of 0	0.380	0.388	99.01
Filling of last data	0.382	0.389	98.93
Filling of mean	0.381	0.389	98.95

### Evaluation Index

To evaluate the prediction performance of the model, the average absolute error *y*_*MAE*_, root mean square error *y*_*RMSE*_, and prediction accuracy *y*_*FA*_ are used as evaluation criteria, and the formulas are defined as follows:


(6) $$\begin{array}{l} y_{\text{MAE}}=\frac{1}{N}\overset{N}{\underset{j=1}{\sum }}|x_{real}\left(j\right)-x_{pred}\left(j\right)| \end{array}$$

(7) $$\begin{array}{l} y_{\text{RMSE}}=\sqrt{\frac{\overset{N}{\underset{j=1}{\sum }}{\left(x_{real}\left(j\right)-x_{pred}\left(j\right)\right)}^{2}}{N}} \end{array}$$

(8) $$\begin{array}{l} y_{\text{FA}}=\frac{1}{N}\overset{N}{\underset{j=1}{\sum }}\left(1-\frac{\left|x_{real}\left(j\right)-x_{pred}\left(j\right)\right|}{x_{real}\left(j\right)}\right)\times \;100\% \end{array}$$

In the formula, *x*_*real*_(*j*) is the *j*th true test data; *x*_*pred*_(*j*) is the *j*th prediction data; and *N* is the number of predictions. When the *y*_*MAE*_ and *y*_*RMSE*_ values are smaller and the *y*_*FA*_ values are larger, the residual magnetic field strength representing the predicted water and fertilizer liquid is closer to the true measured value, that is, the prediction accuracy is higher.

### Experimental Results and Comparison

During model training, antagonistic samples are generated by the *f*_*PGD*_ attack algorithm, which improves the model's defense success rate against antagonistic samples, enhances the game antagonism between generator and the discriminator, improves the generalization ability of the model, and enables CGAN to generate series data closer to the true sample distribution.

**Figure 7:**
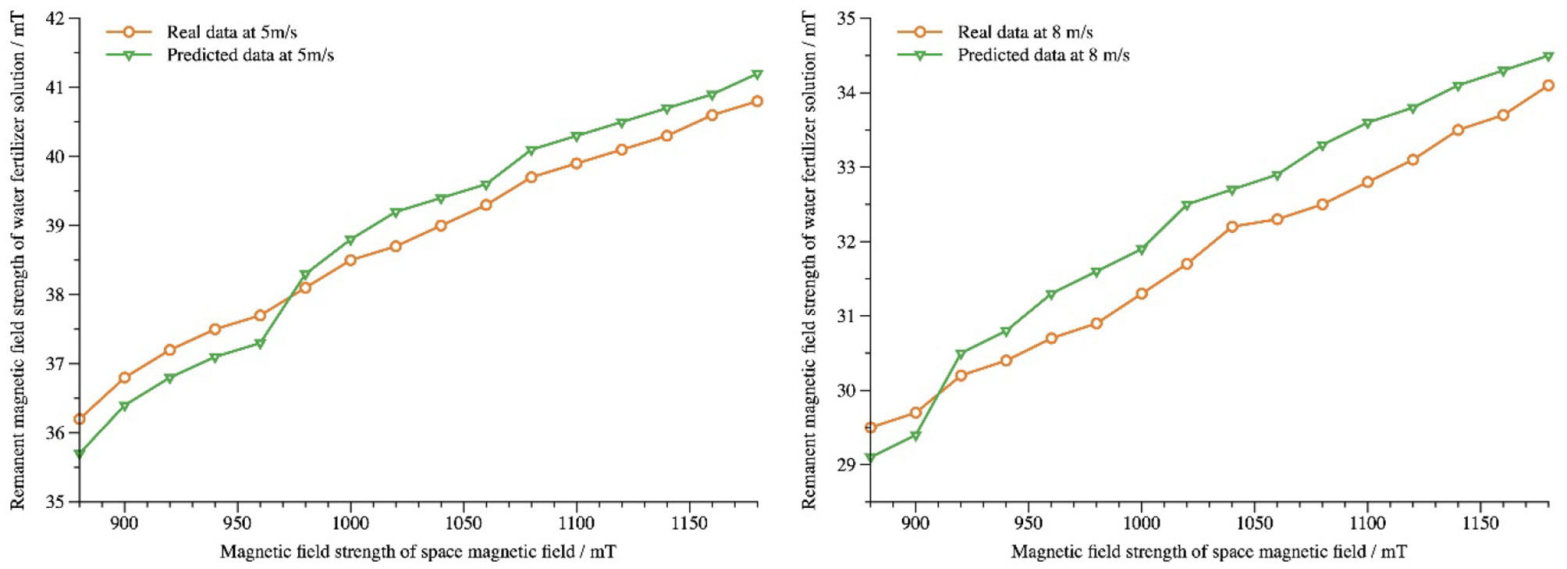
Comparison of predicted data with real detection data.

In this study, the dataset is divided twice differently (Table 3). Residual magnetic field strength data of liquid passing through magnetized space pipeline at speeds 4*m*/*s* and 6*m*/*s* are selected for training dataset. Residual magnetic field strength data at velocity 5*m*/*s* and 8*m*/*s* are used for testing dataset, 16 prediction data are generated, and real detection data are compared for performance evaluation. The comparison between predicted and real data for flow velocities 5*m*/*s* and 8*m*/*s* is shown in Figure 7. From the graph, the prediction model can capture the distribution of real data and predict it well.

Calculate the *y*_*MAE*_, *y*_*RMSE*_, and *y*_*FA*_ values of the predicted data of dynamic water and fertilizer liquid under the magnetization condition of spatial magnetic field strength $880mT\tilde{\;}1180mT$ (equal difference 20*mT*) at flow rates 5*m*/*s* and 8*m*/*s*, respectively, as shown in Table 5. It can be seen from the table that the model can accurately predict the residual magnetic field strength of dynamic water and fertilizer liquid.

**Table 5:** Error and precision comparison of predicted data.

**Velocity of the liquid**	**ȳ_*MAE*_/*mT***	**ȳ_*RMSE*_/*mT***	**ȳ_*FA*_/*%***
5m/s	0.380	0.388	99.01
8m/s	0.570	0.592	98.22

**Figure 8:**
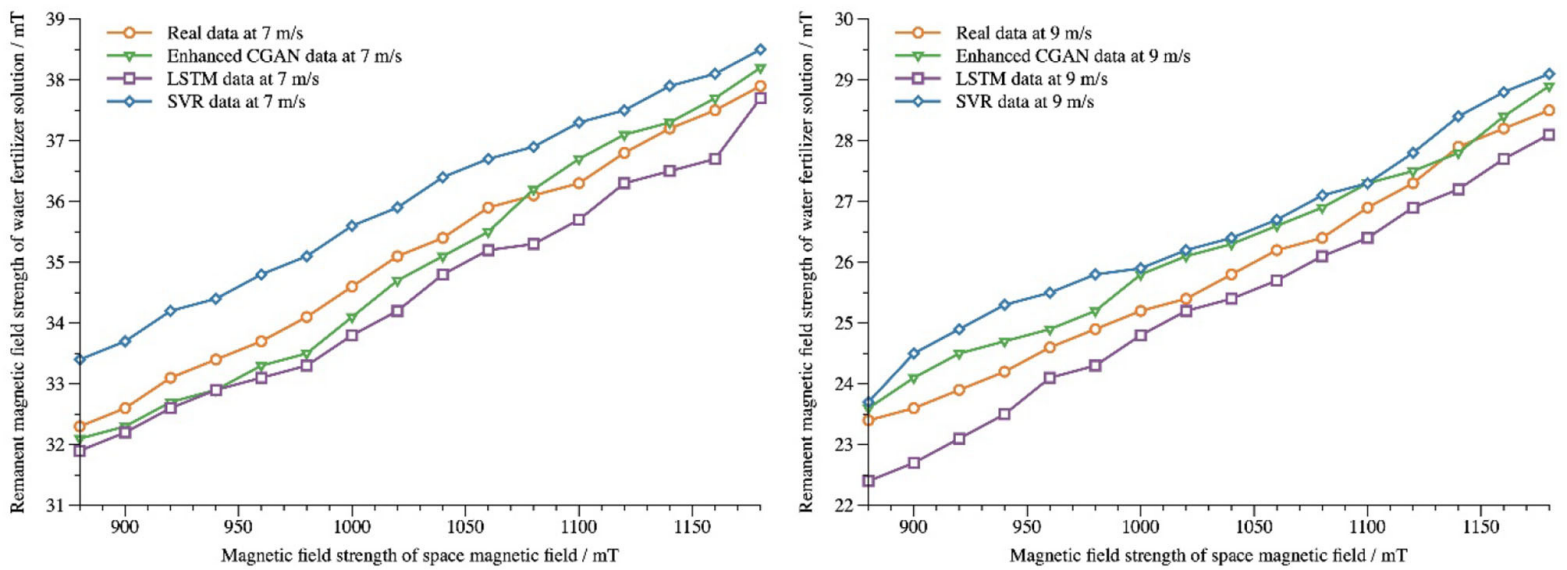
Comparison of prediction results of different prediction models.

Residual magnetic field strength data from query datasets 7*m*/*s* and 9*m*/*s* are tested and compared with LSTM and SVR models with optimized parameters. The parameters of the LSTM model are set to a learning rate of 0.01, a number of iterations of 1,000, 128 cells in the first hidden layer, and 64 cells in the second hidden layer. The parameters of the SVR model are set to radial basis function kernel, penalty factor *C* = 10, and kernel parameter *Y* = 0.25. Figure 8 shows the predicted data of residual magnetic field strength of water and fertilizer liquids at 7*m*/*s*and 9*m*/*s* flow rates and the comparison of predicted results of different prediction models. From the diagram, it can be seen that all three models can make a good prediction of the trend of residual magnetic field strength. The predicted results of the models presented in this paper have the highest coincidence with the actual measured data, and the results are the best.

When the flow rate of water and fertilizer liquid is 7*m*/*s*, the prediction errors and precision of several models are compared under the influence of different spatial magnetic field strength in air gap as shown in Table 6. It can be seen that the average absolute error and root mean square error of the model presented in this paper are the smallest and the prediction precision is the highest compared with those of the other two models for the strength of the gap spatial magnetic field in different intervals.

**Table 6:** Comparison of prediction error and precision under different magnetization conditions (7*m*/*s* velocity).

**Model**	$880mT^{∼}980mT$			$1000mT^{∼}1080mT$			$1100mT^{∼}1180mT$			**Mean**		
	**ȳ_*MAE*_/*mT***	**ȳ_*RMSE*_/*mT***	**ȳ_*FA*_*%***	**ȳ_*MAE*_/*mT***	**ȳ_*RMSE*_/*mT***	**ȳ_*FA*_/*%***	**ȳ_*MAE*_/*mT***	**ȳ_*RMSE*_/*mT***	**ȳ_*FA*_/*%***	**ȳ_*MAE*_/*mT***	**ȳ_*RMSE*_/*mT***	**ȳ_*FA*_/*%***
Enhance CGAN	0.40	0.42	98.8	0.34	0.37	99.0	0.26	0.28	99.3	0.33	0.36	99.0
LSTM	0.53	0.55	98.4	0.76	0.77	97.9	0.56	0.60	98.5	0.62	0.64	98.2
SVR	1.07	1.07	96.8	0.88	0.89	97.5	0.72	0.73	98.1	0.89	0.90	97.5

## Discussion

For current prediction algorithms, researchers pay more attention to time-series data, while there are a large number of series data with weak correlation with time in agricultural production, such as soil humidity and salinity, PH value, and surface tension of irrigation water. These series data are often influenced by a variety of factors, but once the major variables are identified, they change in the same rhythm as time series. Therefore, it is a meaningful attempt to use the machine learning model applied to time-series data prediction to study other series data prediction. This will help to improve the ability of data mining based on the limited detection series data.

In order to fully verify the superiority of the proposed model, we further compare the prediction errors and accuracy of different prediction models for magnetized liquid series data. Long short-term memory (LSTM) neural network is widely used in time-series data prediction, such as weather forecast (Karevan and Suykens, 2020), finance (Cao et al., 2019), and oil production forecast (Song et al., 2020). The architecture of LSTM makes it easy to capture patterns in series data. It has the advantage of being able to learn and remember long sequences and does not rely on pre-specified window observations as input. Therefore, LSTM tends to do a better job of predicting unstable time series with more fixed components. In reference (Maldonado et al., 2019), a power load forecasting method based on Support Vector Regression (SVR) is introduced by Maldonado et al. The method follows the backward variable elimination process based on gradient descent optimization and adjusts the width of the anisotropic Gaussian kernel iteratively. In the model comparison of this paper, the model structure of LSTM and SVR is used for reference to predict the series data of magnetized liquid. The comparison results show that the enhanced CGAN model proposed in this paper has better performance in terms of data. On the one hand, the addition of antagonistic samples in this model improves the performance of discriminator and promotes the generator to generate better data. On the other hand, the real training data are taken as the conditional input of the model so that the model can capture the real data distribution. However, it is undeniable that, as a comparative model, this study did not carry out detailed parameter optimization for LSTM and SVR models, but simply set parameters. At the same time, because LSTM combines long-term memory with short-term memory, it selectively forgets some secondary information and captures the main characteristics of data distribution, so its performance in this experiment is better than that of SVR model.

Because the proposed enhanced CGAN model can further improve the prediction accuracy of liquid magnetization series data, it can help agricultural production managers to better adjust the spatial magnetic field strength during water and fertilizer magnetization irrigation, so that the magnetic biological effect produced by water and fertilizer liquid is more conducive to crop growth and improve its yield and quality. In this study, the technology of anti-attack defense and CGAN generation of prediction data was introduced into the field of liquid magnetization series data prediction, in order to improve the precision of integrated management of agricultural water and fertilizer. Agricultural producers will benefit more if real-time data acquisition technology and computing power of field hardware can be improved. However, the internal network of CGAN in this study only adopts a CNN sub-model. In the case of relatively dense series data, the prediction accuracy is not improved much, and there are still some limitations.

## Conclusion

On the basis of a small amount of detection data, how to make accurate data prediction and provide basis for future decision-making is of great significance to producers and researchers. we propose to combine PGD attack algorithm with CGAN to predict the series data in order to obtain the true data distribution as possible. On the one hand, the limited series data are expanded, and on the other hand, the generated antagonistic samples are used to improve the defense success rate of discriminator in CGAN, so as to guide generator to generate more conditionally distributed data. Experiments show that the proposed prediction model can accurately predict the residual magnetic field strength of water and fertilizer liquids. By comparing with other prediction models, it is also proved that the model proposed in this paper has advantages in prediction precision.

This paper evaluates model performance based on point-by-point error, which is incomplete, because the point-by-point error measurement does not fully reflect the distribution similarity between the predicted data and the real data. At the same time, the dataset used has a single trend. Subsequent work will analyze the characteristics of the detected data under complex changing trends, build comprehensive and accurate evaluation index to reproduce the data distribution, and further improve the prediction precision and universality of the model.

## Data Availability Statement

The raw data supporting the conclusions of this article will be made available by the authors, without undue reservation.

## Author Contributions

JN: methodology and writing—original draft. NW: conceptualization and software. JL: writing—review. YW: visualization. KW: editing. All authors read and approved the final manuscript.

## Funding

This work was supported by the National Natural Science Foundation of China (No. 31860333).

## Conflict of Interest

The authors declare that the research was conducted in the absence of any commercial or financial relationships that could be construed as a potential conflict of interest.

## Publisher's Note

All claims expressed in this article are solely those of the authors and do not necessarily represent those of their affiliated organizations, or those of the publisher, the editors and the reviewers. Any product that may be evaluated in this article, or claim that may be made by its manufacturer, is not guaranteed or endorsed by the publisher.
